# Psychiatric morbidity, somatic comorbidity and substance use in an adolescent psychiatric population at 3-year follow-up

**DOI:** 10.1007/s00787-020-01602-8

**Published:** 2020-07-15

**Authors:** Kari Skulstad Gårdvik, Marite Rygg, Terje Torgersen, Stian Lydersen, Marit Sæbø Indredavik

**Affiliations:** 1 Department of Mental Health, Faculty of Medicine and Health Sciences, Regional Centre for Child and Youth Mental Health and Child Welfare, Norwegian University of Science and Technology, Trondheim, Norway; 2 Division of Mental Health Care, Department of Children and Youth, St. Olavs Hospital, Trondheim University Hospital, Trondheim, Norway; 3 Department of Clinical and Molecular Medicine (IKOM), Faculty of Medicine and Health Sciences, Norwegian University of Science and Technology, Trondheim, Norway; 4 Department of Pediatrics, St. Olavs Hospital, Trondheim University Hospital, Trondheim, Norway; 5 Division of Mental Health Care, Department of Østmarka, St. Olavs Hospital, Trondheim University Hospital, Trondheim, Norway; 6 Department of Mental Health, Faculty of Medicine and Health Sciences, Norwegian University of Science and Technology, Trondheim, Norway

**Keywords:** Mental disorders, Adolescent, Pain, Comorbidity, Longitudinal study

## Abstract

Knowledge is scarce on the course of psychiatric disorders in adolescence. We aimed to assess changes in the frequency of psychiatric disorders, somatic disorders, pain, and substance use in a clinical psychiatric cohort from adolescence to young adulthood. This study is part of the Health Survey in Department of Children and Youth, St. Olavs Hospital, Norway. At age 13–18 years, 717 (43.5% of eligible) participated in the first study visit (*T*_1_) in 2009–2011, 549 were reassessed 3 years later with telephone interview (*T*_2_), and 464 had diagnostic evaluation at both time points. Data included: ICD-10 diagnoses (*T*_1_), DSM-IV diagnoses (*T*_2_), self-reported pain and substance use (*T*_1_ and *T*_2_). The overall rate of psychiatric disorders decreased (*T*_1_ vs. *T*_2_: 94.8% vs. 72.2%, *p* < 0.001); while, an increased rate of anxiety disorders was marked among girls (37.5% vs. 55.9%, *p* < 0.001), with accompanying raised frequencies of psychiatric comorbidity (14.1% vs. 42.6%, *p* < 0.001), somatic comorbidity (9.4% vs. 19.5%, *p* = 0.001), chronic pain (31.6% vs. 49.4%, *p* < 0.001), smoking, alcohol use and trying illicit drugs. Chronic pain, smoking and trying illicit drugs were associated with persisting psychiatric disorders, with highest risk differences for girls (RD = 25.4%, *p* = 0.002, RD = 15.6%, *p* = 0.008, RD = 18.0%, *p* = 0.001, respectively). Three out of four adolescents still had a psychiatric disorder after 3 years. Unlike boys, girls had an increasing rate of anxiety disorders and comorbidities. Chronic pain, smoking and trying illicit drugs were associated with persisting psychiatric disorders. Despite methodological limitations, these findings emphasize the importance of early targeted intervention for adolescents with psychiatric disorders.

**Electronic supplementary material:**

The online version of this article (10.1007/s00787-020-01602-8) contains supplementary material, which is available to authorized users.

## Introduction

An increasing focus on mental disorders in children and adolescents has revealed large variations in prevalence between nations [1]. The worldwide prevalence of mental disorders in this age group was 13.4% in 2015 [2], with anxiety disorders as the most frequent disorder (6.5%). In Norway, the reported prevalence is lower; 8% met the criteria for a psychiatric disorder requiring treatment in 2009 [3, 4]. Still, 15–20% of children and adolescents aged 3–18 years had reduced function due to symptoms of mental disorders [3–5]. Diagnoses differ with age and gender. Before puberty, more boys than girls are diagnosed, and conduct disorder and attention deficit/hyperactivity disorder (ADHD) dominates; while after puberty, the diagnoses and gender predominance shift to anxiety, depression and eating disorders among girls [6, 7]. For both genders, emerging adulthood represents a particularly vulnerable time for the initiation of mental health problems, including substance use [6, 8], and adolescence is the time at which a high burden of disease emerges from mental disorders [9].

Comorbidity of psychiatric disorders is common in children and adolescents, and increases by age [10, 11]. Presence of co-occurring disorders is more marked in girls than in boys [10]. Among adults with psychiatric disorders, almost half have more than one disorder, and comorbidity continues to be more frequent in females [12]. Comorbid psychiatric disorders are challenging to assess and treat, especially in combination with co-existing somatic symptoms or disease [13]. Earlier research have found strong evidence for a relation between somatic symptoms and psychiatric disorders [14] and that psychiatric disorders of all types are associated with an increased risk of onset of a broad range of somatic conditions [15]. A systematic review demonstrated a strong positive association between chronic somatic disorders in adolescence and anxiety and depressive disorders [16]. A recent population-based Swedish study reported a high risk for concurrent somatic disorders in children with psychiatric disorders, across all ages and across many types of conditions [17].

Pain symptoms in adolescence involves an increased risk for mental distress in young adulthood [18, 19], and strong associations are reported between chronic pain and especially anxiety and depression [20]. Therefore, pain seems to be a common symptom and part of the complexity in many psychiatric disorders, especially in anxiety and depressive disorders. In the current sample of interest, when patients with psychiatric disorders were young adolescents, higher rates of chronic pain were found compared to the general adolescent population [21, 22]. In adults with psychiatric disorders, it is well known that chronic somatic conditions are frequent [23]. Still, knowledge is scarce on the longitudinal effect of psychiatric–somatic comorbidity from adolescence to adult age in a clinical psychiatric sample.

Co-existing substance use is another factor contributing to the complexity of mental disorders. Adolescent psychiatric patients have increased risk of substance use [24]. Harmful alcohol consumption combined with depression and anxiety is commonly observed [25, 26]. A recent population-based survey linked with data from National Patient Registry in Norway, found that all investigated psychiatric diagnoses, except autism, were associated with some measure of hazardous alcohol/drug use, with highest odds among adolescents with trauma-related disorders, depression and conduct disorders [27].

Although having a psychiatric disorder in adolescence is a potent risk factor for having a psychiatric disorder in adulthood [28, 29], the frequency of psychiatric disorders is intuitively expected to decline in a clinical follow-up, due to treatment, individual maturation and other factors. However, knowledge is limited on the developmental course of psychiatric morbidity in interplay with co-occurring disorders and substance use in a clinical adolescent cohort. Such knowledge is highly wanted in clinical practice, as a necessary basis for intervention and specific treatment.

The objective of the present study was to examine the prevalence and associations of disorders in a clinical psychiatric cohort over a 3-year period from adolescence to young adulthood. The primary aim was to investigate any changes in the frequency of psychiatric disorders, comorbidities with other psychiatric or somatic disorders, chronic pain, and substance use, overall, by diagnostic groups, and separately for girls and boys. The secondary aim was to study if somatic disorders, chronic pain and substance use were associated with persisting psychiatric disorders, overall, by diagnostic groups, and separately for girls and boys. We hypothesized that the frequency of psychiatric disorders, i.e., anxiety, mood, ADHD and other psychiatric disorders (grouped) declined over the 3 years, and that continuity of a psychiatric disorder was associated with concurrent comorbid disorders, chronic pain and substance use at baseline. We further hypothesized that there would be a different pattern of morbidity for girls and boys.

## Methods

### Study design

The Health Survey in Department of Children and Youth, Division of Mental Health Care, St. Olavs hospital, Trondheim University Hospital, Norway (St. Olav CAP Survey), is a prospective longitudinal cohort study of a defined clinical population assessed at two time points. At time point 1 (*T*_1_), data were collected at inclusion in a cross-sectional study of adolescent patients; at time point 2 (*T*_2_) data were collected at a 3-year follow-up.

At *T*_1_ (2009–2011), all patients aged 13–18 years who visited the Department of Children and Youth (hereafter: CAP clinic) at least once over a 2-year period received both oral and written invitations at their first attendance during the study period. The exclusion criteria were difficulties in answering the survey due to an unstable psychiatric state, low cognitive function, visual impairments, or insufficient language skills. Emergency patients were invited to take part once they entered a stable phase. The participants and their parents received standard application of services. They gave written informed consent to extract diagnostic data from clinical charts and respond to an electronic survey. At *T*_2_ (2012–2014), age 16–21 years, data were collected from the *T*_1_ enrolled sample and their parents, by an electronic survey and a diagnostic telephone interview performed by trained professionals.

### Participants

**Fig. 1:**
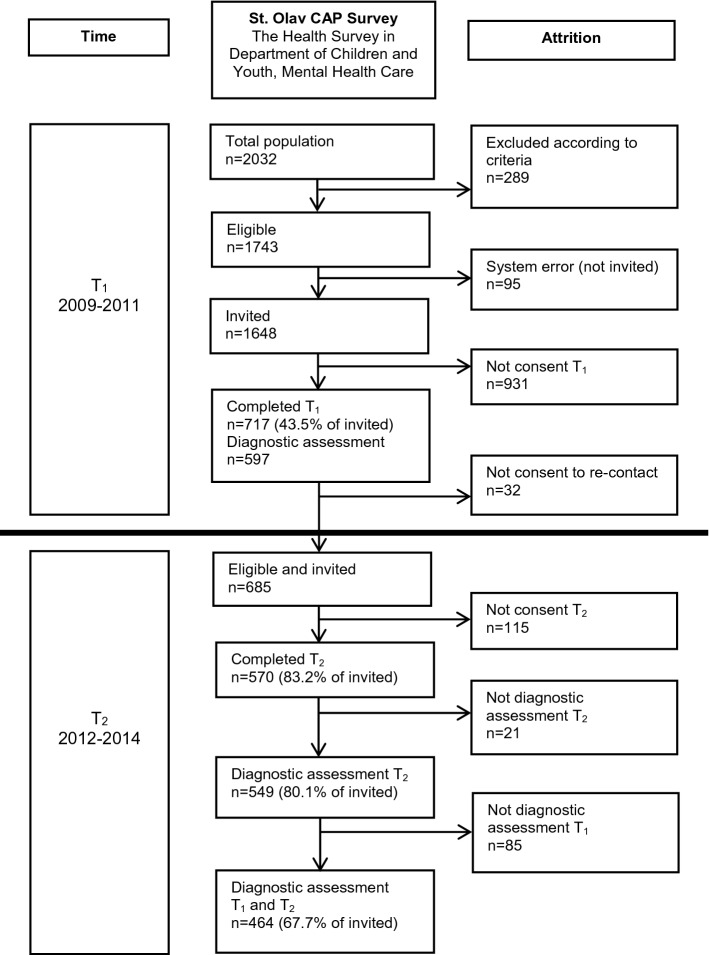
Flow-chart of the recruitment and attrition in the present study

In the *T*_1_ study period, 2032 adolescent patients had at least one attendance at the CAP clinic. Figure 1 demonstrates the participant flow in each stage of the survey. At *T*_1_, *n* = 717 participated (393 (54.8%) girls), of whom *n* = 597 had a complete diagnostic assessment. At *T*_2_, all *T*_1_ participants who previously consented to further inquiry were invited (eligible *n* = 685), of whom 570 (83% of eligible) completed the follow-up questionnaire, and 549 (80%) completed the diagnostic interview (308 (56.1%) girls). The present study included participants with complete diagnostic assessment at both *T*_1_ and *T*_2_ (*n* = 464, 256 (55.2%) girls), with mean age at *T*_1_: 15.7 (range 13.0–20.5), and at *T*_2_: 18.7 (16.0–23.5) years (Table 1).

**Table 1:** Age of participants in diagnostic groups at *T*_1_ and *T*_2_

	Any psychiatric disorder				Anxiety disorders				Mood disorders				ADHD				Other psychiatric disorders			
	*T*_1_		*T*_2_		*T*_1_		*T*_2_		*T*_1_		*T*_2_		*T*_1_		*T*_2_		*T*_1_		*T*_2_	
Total (*n* = 464)																				
Age (years) Mean (SD)	15.7	(1.7)	18.7	(1.7)	15.8	(1.6)	19.0	(1.6)	16.4	(1.5)	19.0	(1.5)	15.4	(1.7)	18.5	(1.7)	15.5	(1.7)	18.8	(1.6)
Girls (*n* = 256)																				
Age (years) Mean (SD)	16.0	(1.7)	19.0	(1.6)	16.0	(1.6)	19.2	(1.6)	16.5	(1.5)	19.0	(1.4)	15.9	(1.9)	18.8	(1.7)	16.1	(1.7)	19.3	(1.6)
Boys (*n* = 208)																				
Age (years) Mean (SD)	15.3	(1.6)	18.3	(1.6)	15.4	(1.6)	18.5	(1.7)	15.9	(1.6)	18.8	(1.8)	15.2	(1.5)	18.2	(1.6)	15.1	(1.6)	18.3	(1.6)

### Participants vs. non-participants

To explore the representativeness of the study population at *T*_1_, anonymous information about the total clinical population was collected from annual reports from the CAP clinic, 2009–2011. All adolescents in the study period (*n* = 2032) minus those excluded (*n* = 289) were defined as reference population (*n* = 1743). The main reason for referral, age and gender were similar between participants (*n* = 717, 41.1%) and non-participants (*n* = 1026, 58.9%) (data not shown). Participants were 0.27 years older: mean (SD) 15.7 (1.7) vs. 15.4 (2.0), and there were more girls among the participants: 393 (54.8%) vs. 509 (49.6%). Among those with complete diagnostic assessment at *T*_1_, there were 464 participants and 133 non-participants at *T*_2_. Attrition analyses are given in Supplementary Material (Tables S1, S2 and S3). Age, socioeconomic status and frequencies of any psychiatric disorder were similar among participants and non-participants; while, the proportion of girls was higher among participants.

### Measures

Psychiatric diagnoses at *T*_1_ were set in ordinary clinical practice according to the International Statistical Classification of Disease and Related Health Problems (ICD-10) multiaxial diagnostics (axes I–VI) [30]. The diagnostic process followed standardized procedures for assessment and diagnosis of common adolescent psychiatric disorders, requiring a thorough developmental history and interviews with the adolescents and their parents. For some participants, the semi-structured Schedule for Affective Disorders and Schizophrenia for School-Age Children (K-SADS) [31] was used, for others the Development And Well-Being Assessment (DAWBA) [32], and various rating scales suitable for the presenting problem. The diagnoses were set by a child and adolescent psychiatrist or a clinical psychologist based on all available clinical information, after consensus with other professionals from the multi-disciplinary team. The assessments were supplemented with somatic examination if indicated, and possible coexisting disorders were explored. At *T*_2_, diagnoses were set using the K-SADS [31] according to the Diagnostic and Statistical Manual of Mental Disorders IV Text revision (DSM-IV-TR) [33]. The interviews were performed with the adolescents by telephone by trained interviewers, all with a graduate degree in medicine or psychology and experience in child and adolescent psychiatric assessment. They met regularly with a supervisor, an experienced child and adolescent psychiatrist, to assure the quality of the diagnostic assessment. All were blinded to *T*_1_ diagnoses. Inter-rater reliability was assessed using second ratings for 28 of the taped telephone interviews. Because of weaknesses of kappa as measure of agreement [34], positive and negative agreement were used as measurement. Positive agreement, as defined by van de Vet et al., varied from 0.615 to 1.000 and, negative agreement varied from 0.884 to 1.000. Details are given in Supplementary Material (Tables S4 and S5).

In the present study, disorders were grouped into the following categories, based on ICD-10 diagnoses at *T*_1_ and DSM-IV diagnoses at *T*_2_; any psychiatric disorder, anxiety disorders (ICD-codes F40-F44, F48, F93/DSM-codes 300, 308, 309), mood disorders (ICD-codes F31-F34, F38, F39/DSM-codes 296, 300.4, 311), ADHD (ICD-code F90/DSM-code 314) and other (ICD-codes F10-F19, F20-F21, F28-F29, F50, F54, F59-F60, F84, F91-F92, F94-F95, F98/DSM-codes 291, 292, 295, 298, 299, 301, 303, 304, 305, 307, 312, 313, 316). As there were few participants in some diagnostic groups, for example autism and eating disorders, and especially when examining comorbidity, we chose to merge children with these diagnoses into one larger group of other psychiatric disorders for the purpose of this manuscript.

Somatic disorders at *T*_1_ were registered according to ICD-10 axis 4, set by the medical doctor based on anamnestic information, the entire medical records, including pediatrics, or by clinical investigation. All patients reporting somatic symptoms or disorders had an evaluation by a medical doctor. At *T*_2_, somatic disorders were recorded as part of the K-SADS interview. Somatic comorbidity was defined as having a psychiatric disorder with a co-occurring somatic disorder requiring regular clinical follow-ups.

Chronic pain (*T*_1_ and *T*_2_) was defined as the pain not related to any known disease or injury, occurring at least once a week in the last 3 months. The test–retest reliability of questions of pain occurrence at least once a week for the last 3 months has shown to be good [20, 35]. As in previous studies, adolescents were asked to fill in a questionnaire and specify if they had experienced headaches, abdominal pain or musculoskeletal pain (e.g., pain in the neck, shoulder, upper and lower extremities, upper back, lower back/seat or chest), accompanied with an illustration of the different locations [20, 22]. Multisite pain was defined as having chronic pain in three locations or more [20, 22].

Substance use was registered by self-report as smoking, alcohol use and drug use. “Current smokers” included daily or occasional smokers (*T*_1_ and *T*_2_). “Current alcohol users” included participants, who answered “yes” to the following questions: *T*_1_: “Do you sometimes drink alcohol presently?”, and *T*_2_: “Have you drunk alcohol during the last four weeks?”. “Drug use” was indicated by answering “yes” to the question: “Have you ever tried hash, marijuana, or other illicit drugs?” (*T*_1_ and *T*_2_).

Socioeconomic status (SES) was measured at *T*_1_ by the mothers’ highest level of education, divided into eight categories: (1) less than 9-year primary school; (2) completed 9-year primary school; (3) 1 or 2 years in high school; (4) completed high school; (5) completed high school and 1-year education/training after high school; (6) academy/university for up to and including 4 years; (7) academy/university for 5 years or more; and (8) academy/university including PhD.

### Statistical analyses

The change in point prevalence from *T*_1_ to *T*_2_ was based on paired dichotomous data, with Newcombe confidence intervals and the McNemar asymptotic test, as recommended by Fagerland, Lydersen and Laake [36]. We used binary linear regression with psychiatric disorder at *T*_2_ as dependent variable and relevant variables at *T*_1_, one at a time, to study their associations. Effects of age and SES as possible confounders were explored. We reported 95% confidence intervals (CI) where relevant, and two-sided *p* values < 0.05 were considered statistically significant. Binary regression was performed in Stata 15, Newcombe CI and McNemars test were calculated in Excel, and the rest in SPSS 25.

### Ethics

Written informed consent was obtained from adolescents and parents prior to inclusion at *T*_1_, and from the adolescents at *T*_2_, according to study procedures. Study approval was given by the Regional committee for Medical and Health Research Ethics (reference numbers CAP survey *T*_1_: 4.2008.1393, *T*_2_: 2011/1435/REK Midt, and present study using *T*_1_ and *T*_2_ data: 2017/589/REK Midt). The Norwegian Social Science Data Services, The Data Protection Official for Research, gave permission to compare the main reason for referral, age and sex between participants and non-participants in connection with inclusion at baseline (reference number CAP survey: 19976).

## Results

### Psychiatric disorders and comorbidity

The overall rate of diagnoses decreased from 94.8% at *T*_1_ to 72.2% at *T*_2_. The change [risk difference; RD = − 22.6%, CI (− 26.9, − 18.5), *p* < 0.001] was present in both genders (Table 2). However, the frequency of anxiety disorders increased in the total sample [31.7% vs. 40.1%, RD = 8.4%, CI (2.7, 14.0), *p* = 0.004], but among girls only [37.5% vs. 55.9%, RD = 18.4%, CI (10.1, 26.3), *p* < 0.001]. Psychiatric comorbidity increased in any psychiatric disorder [28.0% vs. 36.4%, RD = 8.4%, CI (2.8, 13.9), *p* = 0.003], but for girls only [26.6% vs. 45.3%, RD = 18.7%, CI (10.5, 26.6), *p* < 0.001]. The increase in psychiatric comorbidity in the total sample was statistically significant in anxiety disorders, mood disorders and ADHD, and in all diagnostic subgroups for girls, but not for boys (Table 2).

**Table 2:** Changes in frequencies of psychiatric disorders and comorbid psychiatric disorders from *T*_1_ to *T*_2_

	Any psychiatric disorder			Anxiety disorders			Mood disorders			ADHD^a^			Other psychiatric disorders		
	*T*_1_	*T*_2_	RD^b^ % (95% CI) *p* value	*T*_1_	*T*_2_	RD % (95% CI) *p* value	*T*_1_	*T*_2_	RD % (95% CI) *p* value	*T*_1_	*T*_2_	RD % (95% CI) *p* value	*T*_1_	*T*_2_	RD % (95% CI) *p* value
Total (*n* = 464)															
Psychiatric disorder															
*n*/*n*_total_^c^ (%)	440/464 (94.8)	335/464 (72.2)	− 22.6 (− 26.9, − 18.5) < 0.001	147/464 (31.7)	186/464 (40.1)	8.4 (2.7, 14.0) 0.004	99/464 (21.3)	77/464 (16.6)	− 4.7 (− 9.1, − 0.4) 0.031	204/463 (44.0)	192/463 (41.5)	− 2.5 (− 6.8, 1.7) 0.230	120/464 (25.9)	107/464 (23.1)	− 2.8 (− 7.2, 1.6) 0.209
Comorbid psychiatric disorder															
*n*/*n*_disorder_^d (^%) *n*/*n*_total_^e^ (%)	130/440 (29.5)	169/335 (50.5)		59/147 (40.1)	136/186 (73.1)		45/99 (45.5)	73/77 (94.8)		75/204 (36.8)	100/192 (52.1)		70/120 (58.3)	86/107 (80.4)	
	130/464 (28.0)	169/464 (36.4)	8.4 (2.8, 13.9) 0.003	59/464 (12.7)	136/464 (29.3)	16.6 (11.6, 21.1) < 0.001	45/464 (9.7)	73/464 (15.7)	6.0 (2.0, 10.1) 0.004	75/463 (16.2)	100/463 (21.6)	5.4 (1.0, 9.8) 0.020	70/464 (15.1)	86/464 (18.5)	3.4 (− 0.7, 7.6) 0.100
Girls (*n* = 256)															
Psychiatric disorder															
*n*/*n*_total_ (%)	245/256 (95.7)	191/256 (74.6)	− 21.1 (− 26.7, − 15.8) < 0.001	96/256 (37.5)	143/256 (55.9)	18.4 (10.1, 26.3) < 0.001	82/256 (32.0)	66/256 (25.8)	− 6.2 (− 13.1, 0.7) 0.077	83/255 (32.6)	91/255 (35.7)	3.1 (− 2.1, 8.4) 0.238	52/256 (20.3)	50/256 (19.5)	− 0.8 (− 6.6, 5.0) 0.789
Comorbid psychiatric disorder															
*n*/*n*_disorder (_%) *n*/*n*_total_ (%)	68/245 (27.8)	116/191 (60.7)		36/96 (37.5)	109/143 (76.2)		38/82 (46.3)	62/66 (93.9)		29/83 (34.9)	59/91 (64.8)		23/52 (44.2)	45/50 (90.0)	
	68/256 (26.6)	116/256 (45.3)	18.7 (10.5, 26.6) < 0.001	36/256 (14.1)	109/256 (42.6)	28.5 (20.8, 35.8) < 0.001	38/256 (14.8)	62/256 (24.2)	9.4 (2.8, 15.9) 0.005	29/255 (11.4)	59/255 (23.1)	11.7 (5.9, 17.7) < 0.001	23/256 (9.0)	45/256 (17.6)	8.6 (3.2, 14.1) 0.002
Boys (*n* = 208)															
Psychiatric disorder															
*n*/*n*_total_ (%)	195/208 (93.8)	144/208 (69.2)	− 24.6 (− 31.1, − 18.0) < 0.001	51/208 (24.5)	43/208 (20.7)	− 3.8 (− 11.2, 3.5) 0.302	17/208 (8.2)	11/208 (5.3)	− 2.9 (− 7.7, 1.8) 0.201	121/208 (58.2)	101/208 (48.6)	− 9.6 (− 16.3, − 2.7) 0.007	68/208 (32.7)	57/208 (27.4)	− 5.3 (− 12.0, 1.5) 0.124
Comorbid psychiatric disorder															
*n*/*n*_disorder (_%) *n*/*n*_total_ (%)	62/195 (31.8)	53/144 (36.8)		23/51 (45.1)	27/43 (62.8)		7/17 (41.2)	11/11 (100)		46/121 (38.0)	41/101 (40.6)		47/68 (69.1)	41/57 (71.9)	
	62/208 (29.8)	53/208 (25.5)	− 4.3 (− 11.4, 2.8) 0.233	23/208 (11.1)	27/208 (13.0)	1.9 (− 3.6, 7.5) 0.480	7/208 (3.4)	11/208 (5.3)	1.9 (− 2.3, 6.3) 0.346	46/208 (22.1)	41/208 (19.7)	− 2.4 (− 9.0, 4.1) 0.466	47/208 (22.6)	41/208 (19.7)	− 2.9 (− 9.2, 3.5) 0.366

Newcombe confidence intervals and McNemar asymptotic *p* values are based on paired data displayed in Supplemental Material Table S6

Psychiatric disorder includes both primary and additional diagnoses, based on only complete case (diagnostic assessment at both *T*_1_ and *T*_2_)

Comorbid psychiatric disorder includes all patients with more than one psychiatric disorder

^a^In ADHD group there is 1 missing among girls

^b^RD is risk difference, the difference between the proportions of patients with psychiatric disorder or comorbid psychiatric disorder at *T*_2_ compared with *T*_1_

^c^*n*/*n*_total_ means that there are (*n*) numbers of patients with psychiatric disorder among the total sample of patients

^d^*n*/*n*_disorder_ means that there are (*n*) numbers of patients with comorbid psychiatric disorder among the patients with the actual psychiatric disorder

^e^*n*/*n*_total_ means that there are (*n*) numbers of patients with comorbid psychiatric disorder among the total sample of patients

### Somatic comorbidity and chronic pain

Somatic comorbidity increased in frequency in the anxiety disorder group [7.1% vs. 12.7%, RD = 5.6%, CI (2.0, 9.3), *p* = 0.003], but only among girls [9.4% vs. 19.5%, RD = 10.1%, CI (4.3, 16.1), *p* = 0.001] (Table 3). For other psychiatric disorders, somatic comorbidity remained unchanged in both genders. The prevalence of chronic pain in the cohort was high, but decreased overall in the total sample [65.8% vs. 49.3%, RD = − 16.5%, CI (− 21.0, − 11.0), *p* < 0.001], and for both girls and boys. However, chronic pain increased among patients with anxiety disorders [23.3% vs. 31.8%, RD = 8.5%, CI (3.1, 13.7), *p* = 0.002], but the increase was found only among girls [31.6% vs. 49.4%, RD = 17.9%, CI (9.5, 25.4), *p* < 0.001]. At *T*_2_, girls with anxiety disorders had a higher rate of chronic pain than boys with these disorders (88.7% vs. 48.8%), and also a higher rate of multisite pain (66.2% vs. 20.9%). The diagnostic group with highest frequencies of chronic pain was mood disorders for both girls (96.9%) and boys (72.7%). The frequencies of multisite pain were also highest in this diagnostic group.

**Table 3:** Changes in frequencies of psychiatric disorders with comorbid somatic disorders and pain from *T*_1_ to *T*_2_

	Any psychiatric disorder			Anxiety disorders			Mood disorders			ADHD			Other psychiatric disorders		
	*T*_1_	*T*_2_	RD^a^ % (95% CI) *p* value	*T*_1_	*T*_2_	RD % (95% CI) *p* value	*T*_1_	*T*_2_	RD % (95% CI) *p* value	*T*_1_	*T*_2_	RD % (95% CI) *p* value	*T*_1_	*T*_2_	RD % (95% CI) *p* value
Total (*n* = 464)															
Comorbid somatic disorder															
*n*/*n*_disorder_^b (^%) *n*/*n*_total_^c (^%)	81/440 (18.4)	85/335 (25.4)		33/147 (22.5)	59/186 (31.7)		21/99 (21.2)	24/77 (31.2)		31/203 (15.3)	46/192 (24.0)		20/120 (16.7)	27/107 (25.2)	
	81/464 (17.5)	85/464 (18.3)	0.8 (− 3.8, 5.5) 0.713	33/464 (7.1)	59/464 (12.7)	5.6 (2.0, 9.3) 0.003	21/464 (4.5)	24/464 (5.2)	0.7 (− 2.0, 3.3) 0.612	31/463 (6.7)	46/463 (9.9)	3.2 (− 0.2, 6.7) 0.059	20/464 (4.3)	27/464 (5.8)	1.5 (− 1.3, 4.4) 0.274
Chronic pain^d^															
*n*/*n*_disorder_ (%) *n*/*n*_total_ (%)	304/438 (69.4)	225/330 (68.2)		108/147 (73.5)	147/185 (79.5)		79/99 (79.8)	71/76 (93.4)		135/202 (66.8)	122/188 (64.9)		74/120 (61.7)	70/106 (66.0)	
	304/462 (65.8)	225/456 (49.3)	− 16.5 (− 21.0, − 11.0) < 0.001	108/463 (23.3)	147/463 (31.8)	8.5 (3.1, 13.7) 0.002	79/464 (17.0)	71/463 (15.3)	− 1.7 (− 5.8, 2.3) 0.400	135/462 (29.2)	122/461 (26.5)	− 2.7 (− 7.2, 2.0) 0.261	74/464 (16.0)	70/463 (15.1)	− 0.9 (− 4.6, 3.3) 0.745
Multisite pain^e^															
*n*/*n*_disorder_ (%) *n*/*n*_total_ (%)	171/435 (39.3)	141/329 (42.9)		68/145 (46.9)	103/185 (55.7)		56/99 (56.6)	51/76 (67.1)		71/201 (35.3)	67/187 (35.8)		39/120 (32.5)	45/106 (42.5)	
	171/459 (37.3)	141/455 (31.0)	− 6.3 (− 13.3, − 2.1) 0.008	68/462 (14.7)	103/463 (22.3)	7.6 (3.1, 12.1) 0.001	56/464 (12.1)	51/463 (11.0)	− 1.1 (− 4.7, 2.5) 0.553	71/461 (15.4)	67/462 (14.5)	− 0.9 (− 4.5, 3.1) 0.732	39/464 (8.4)	45/463 (9.7)	1.3 (− 1.7, 4.3) 0.386
Girls (*n* = 256)															
Comorbid somatic disorder															
*n*/*n*_disorder_ (%) *n*/*n*_total_ (%)	53/245 (21.6)	57/191 (29.8)		24/96 (25.0)	50/143 (35.0)		17/82 (20.7)	22/66 (33.3)		14/83 (16.9)	25/91 (27.5)		11/52 (21.2)	18/50 (36.0)	
	53/256 (20.7)	57/256 (22.3)	1.6 (− 5.1, 8.2) 0.642	24/256 (9.4)	50/256 (19.5)	10.1 (4.3, 16.1) 0.001	17/256 (6.6)	22/256 (8.6)	2.0 (− 2.3, 6.3) 0.353	14/255 (5.5)	25/255 (9.8)	4.3 (− 0.2, 9.0) 0.056	11/256 (4.3)	18/256 (7.0)	2.7 (− 1.1, 6.8) 0.144
Chronic pain															
*n*/*n*_disorder_ (%) *n*/*n*_total_ (%)	202/245 (82.5)	158/189 (83.6)		81/96 (84.4)	126/142 (88.7)		69/82 (84.2)	63/65 (96.9)		69/83 (83.1)	74/89 (83.2)		42/52 (80.8)	41/50 (82.0)	
	202/256 (78.9)	158/254 (62.2)	− 16.7 (− 46.8, − 21.5) < 0.001	81/256 (31.6)	126/255 (49.4)	17.8 (9.5, 25.4) < 0.001	69/256 (27.0)	63/255 (24.7)	− 2.3 (− 9.0, 4.3) 0.486	69/256 (27.0)	74/255 (29.0)	2.0 (− 3.4, 7.3) 0.466	42/256 (16.4)	41/256 (16.0)	− 0.4 (− 5.9, 5.1) 0.886
Multisite pain															
*n*/*n*_disorder_ (%) *n*/*n*_total_ (%)	131/243 (53.9)	114/189 (60.3)		56/94 (59.6)	94/142 (66.2)		53/82 (64.6)	47/65 (72.3)		46/83 (55.4)	49/89 (55.1)		23/52 (44.2)	34/50 (68.0)	
	131/254 (51.6)	114/254 (44.9)	− 6.7 (− 17.6, 0.8) 0.074	56/254 (22.1)	94/255 (36.9)	14.8 (7.7, 22.1) < 0.001	53/256 (20.7)	47/255 (18.4)	− 2.3 (− 8.6, 3.9) 0.453	46/256 (18.0)	49/255 (19.2)	1.2 (− 3.9, 7.1) 0.572	23/256 (9.0)	34/256 (13.3)	4.3 (− 0.2, 8.9) 0.055
Boys (*n* = 208)															
Comorbid somatic disorder															
*n*/*n*_disorder_ (%) *n*/*n*_total_ (%)	28/195 (14.4)	28/144 (19.4)		9/51 (17.6)	9/43 (20.9)		4/17 (23.5)	2/11 (18.2)		17/121 (14.0)	21/101 (20.8)		9/68 (13.2)	9/57 (15.8)	
	28/208 (13.5)	28/208 (13.5)	0 (− 6.4, 6.4) 1	9/208 (4.3)	9/208 (4.3)	0 (− 4.0, 4.0) 1	4/208 (1.9)	2/208 (1.0)	− 0.9 (− 4.0, 1.8) 0.414	17/208 (8.2)	21/208 (10.1)	1.9 (− 3.5, 7.4) 0.465	9/208 (4.3)	9/208 (4.3)	0 (− 4.3, 4.3) 1
Chronic pain															
*n*/*n*_disorder_ (%) *n*/*n*_total_ (%)	102/193 (52.9)	67/141 (47.5)		27/51 (52.9)	21/43 (48.8)		10/17 (58.8)	8/11 (72.7)		66/119 (55.5)	48/99 (48.5)		32/68 (47.1)	29/56 (51.8)	
	102/206 (49.5)	67/202 (33.2)	− 16.3 (− 29.0, − 9.3) < 0.001	27/208 (13.0)	21/208 (10.1)	− 2.9 (− 9.1, 3.3) 0.355	10/208 (4.8)	8/208 (3.9)	− 0.9 (− 5.2, 3.2) 0.617	66/206 (32.0)	48/206 (23.3)	− 8.7 (− 16.1, − 0.5) 0.038	32/208 (15.4)	29/207 (14.0)	− 1.4 (− 6.8, 4.9) 0.739
Multisite pain															
*n*/*n*_disorder_ (%) *n*/*n*_total_ (%)	40/192 (20.8)	27/140 (19.3)		12/51 (23.5)	9/43 (20.9)		3/17 (17.7)	4/11 (36.4)		25/118 (21.2)	18.98 (18.4)		16/68 (23.5)	11/56 (19.6)	
	40/205 (19.5)	27/201 (13.4)	− 6.1 (− 7.4, − 0.4) 0.029	12/208 (5.8)	9/208 (4.3)	− 1.5 (− 6.0, 3.0) 0.491	3/208 (1.4)	4/208 (1.9)	0.5 (− 2.5, 3.6) 0.705	25/205 (12.2)	18/207 (8.7)	− 3.5 (− 8.7, 1.7) 0.178	16/208 (7.7)	11/207 (5.3)	− 2.4 (− 6.6, 1.5) 0.197

Newcombe confidence intervals and McNemar asymptotic *p* values are based on paired data displayed in Supplementary Material Table S7

Psychiatric disorder includes both primary and additional diagnoses, based on only complete case (diagnostic assessment at both *T*_1_ and *T*_2_)

Comorbid somatic disorder includes all patients with somatic disorders that require regular controls

^a^RD is risk difference, the difference between the proportions of patients with psychiatric disorder or comorbid psychiatric disorder at *T*_2_ compared with *T*_1_

^b^*n*/*n*_disorder_ means that there are (*n*) numbers of patients with comorbid somatic disorder among the patients with the actual psychiatric disorder

^c^*n*/*n*_total_ means that there are (*n*) numbers of patients with comorbid somatic disorder among the total sample of patients

^d^Chronic pain was defined as pain occurring at least once a week in the last 3 months, not related to any known disease or injury

^e^Multisite pain was defined as having chronic pain in three locations or more

### Substance use

The amount of substance use changed during the 3-year follow-up (Table 4). There was a non-significant reduction in smoking [30.3% vs. 19.6%, RD = − 10.6%, CI (− 10.4, 0.5), *p* = 0.074] in the total sample, but smoking increased for anxiety disorders [RD = 4.5%, CI (2.0, 9.2), *p* = 0.002], and only among girls. Alcohol use increased for anxiety disorders and ADHD in the total sample [RD = 10.5%, CI (6.3, 16.3), *p* < 0.001, and RD = 7.0%, CI (3.6, 13.1), *p* < 0.001, respectively], and among girls with these disorders. Trying illicit drugs increased overall, in both genders, and in all diagnostic subgroups for girls, with the highest increase in the anxiety group [4.7% vs. 22.4%, RD = 17.7%, CI (12.8, 23.7), *p* < 0.001]. Among boys, an increase was seen in the ADHD group [6.3% vs. 15.1%, RD = 8.8%, CI (5.6, 15.1), *p* < 0.001] and in the group of other psychiatric disorders.

**Table 4:** Changes in frequencies of psychiatric disorders with comorbid substance use from *T*_1_ to *T*_2_

	Any psychiatric disorder			Anxiety disorders			Mood disorders			ADHD				Other psychiatric disorders		
	*T*_1_	*T*_2_	RD^a^ % (95% CI) *p* value	*T*_1_	*T*_2_	RD % (95% CI) *p* value	*T*_1_	*T*_2_	RD % (95% CI) *p* value	*T*_1_	*T*_2_	RD % (95% CI) *p* value	*T*_1_		*T*_2_	RD % (95% CI) *p* value
Total (*n* = 464)																
Smoking^b^ *n*/*n*_disorder_^c^ (%)	99/304 (32.6)	90/330 (27.3)		29/97 (29.9)	53/185 (28.7)		30/75 (40.0)	29/76 (38.2)		46/147 (31.3)	45/188 (23.9)		25/76 (32.9)		35/106 (33.0)	
*n*/*n*_total_^d^ (%)	99/328 (30.2)	90/459 (19.6)	− 10.6 (− 10.4, 0.5) 0.074	29/414 (7.0)	53/463 (11.5)	4.5 (2.0, 9.2) 0.002	30/440 (6.8)	29/463 (6.3)	− 0.5 (− 3.0, 2.6) 0.882	46/407 (11.3)	45/460 (9.8)	− 1.5 (− 4.4, 2.9) 0.680	25/420 (6.0)		35/463 (7.6)	1.6 (− 1.2, 4.7) 0.228
Alcohol use^e^ *n*/*n*_disorder_ (%)	217/440 (49.3)	207/321 (64.5)		71/147 (48.3)	118/180 (65.6)		65/99 (65.7)	46/75 (61.3)		91/204 (44.6)	121/183 (66.1)		58/120 (48.3)		63/102 (61.8)	
*n*/*n*_total_ (%)	217/464 (46.8)	207/450 (46.0)	− 0.8 (− 8.1, 6.9) 0.875	71/464 (15.3)	118/458 (25.8)	10.5 (6.3, 16.3) < 0.001	65/464 (14.0)	46/462 (10.0)	− 4.0 (− 7.8, 0.3) 0.033	91/464 (19.6)	121/455 (26.6)	7.0 (3.6, 13.1) < 0.001	58/464 (12.5)		63/459 (13.7)	1.2 (− 2.6, 5.1) 0.518
Drug use^f^ *n*/*n*_disorder_ (%)	61/437 (14.0)	113/329 (34.4)		18/147 (12.2)	69/185 (37.3)		18/99 (18.2)	35/76 (46.1)		33/201 (16.4)	69/187 (36.9)		15/120 (12.5)		45/105 (42.9)	
*n*/*n*_total_ (%)	61/461 (13.2)	113/458 (24.7)	11.5 (8.3, 16.5) < 0.001	18/464 (3.9)	69/463 (14.9)	11.0 (7.8, 14.7) < 0.001	18/464 (3.9)	35/463 (7.6)	3.7 (0.8, 6.6) 0.011	33/461 (7.2)	69/459 (15.0)	7.8 (5.1, 11.6) < 0.001	15/464 (3.2)		45/462 (9.7)	6.5 (3.6, 9.6) < 0.001
Girls (*n* = 256)																
Smoking *n*/*n*_disorder_ (%)	66/178 (37.1)	56/189 (29.6)		23/66 (34.9)	44/142 (31.0)		25/65 (38.5)	24/65 (36.9)		26/63 (41.3)	20/89 (22.5)		14/39 (35.9)		20/50 (40.0)	
*n*/*n*_total_ (%)	66/189 (34.9)	56/254 (22.1)	− 12.8 (− 14.8, 0.6) 0.069	23/226 (10.2)	44/255 (17.3)	7.1 (3.6, 14.9) 0.001	25/239 (10.5)	24/255 (9.4)	− 1.1 (− 5.2, 4.4) 0.873	26/236 (11.0)	20/254 (7.9)	− 3.1 (− 8.0, 1.1) 0.131	14/243 (5.8)		20/256 (7.8)	2.0 (− 1.8, 5.6) 0.297
Alcohol use *n*/*n*_disorder_ (%)	140/245 (57.1)	130/187 (69.5)		55/96 (57.3)	97/140 (69.3)		57/82 (69.5)	42/64 (65.6)		41/83 (49.4)	61/88 (69.3)		34/52 (65.4)		39/50 (78.0)	
*n*/*n*_total_ (%)	140/256 (54.7)	130/252 (51.6)	− 3.1 (− 15.5, 6.4) 0.414	55/256 (21.5)	97/253 (38.3)	16.8 (10.7, 26.7) < 0.001	57/256 (22.3)	42/254 (16.5)	− 5.8 (− 12.4, 0.6) 0.071	41/256 (16.0)	61/253 (24.1)	8.1 (3.3, 15.1) 0.002	34/256 (13.3)		39/256 (15.2)	1.9 (− 3.5, 7.4) 0.484
Drug use *n*/*n*_disorder_ (%)	37/244 (15.2)	73/189 (38.6)		12/96 (12.5)	57/142 (40.1)		15/82 (18.3)	30/65 (46.2)		20/82 (24.4)	38/89 (42.7)		8/52 (15.4)		29/50 (58.0)	
*n*/*n*_total_ (%)	37/255 (14.5)	73/254 (28.7)	14.2 (9.7, 21.7) < 0.001	12/256 (4.7)	57/255 (22.4)	17.7 (12.8, 23.7) < 0.001	15/256 (5.9)	30/255 (11.8)	5.9 (1.0, 10.6) 0.016	20/255 (7.8)	38/254 (15.0)	7.2 (2.5, 11.8) 0.002	8/256 (3.1)		29/256 (11.3)	8.2 (3.6, 10.9) < 0.001
Boys (*n* = 208)																
Smoking *n*/*n*_disorder_ (%)	33/126 (26.2)	34/141 (24.1)		6/31 (19.4)	9/43 (20.9)		5/10 (50.0)	5/11 (45.5)		20/84 (23.8)	25/99 (25.3)		11/37 (29.7)		15/56 (26.8)	
*n*/*n*_total_ (%)	33/138 (23.9)	34/205 (16.6)	− 7.3 (− 10.1, 5.7) 0.578	6/177 (3.4)	9/208 (4.3)	0.9 (− 3.3, 5.5) 0.593	5/201 (2.5)	5/208 (2.4)	− 0.1 (− 2.6, 2.6) 1	20/171 (11.7)	25/206 (12.1)	0.4 (− 3.1, 9.3) 0.317	11/177 (6.2)		15/207 (7.3)	1.1 (− 3.4, 6.6) 0.513
Alcohol use *n*/*n*_disorder_ (%)	77/195 (39.5)	77/134 (57.5)		16/51 (31.4)	21/40 (52.5)		8/17 (47.1)	4/11 (36.4)		50/121 (41.3)	60/95 (63.2)		24/68 (35.3)		24/52 (46.2)	
*n*/*n*_total_ (%)	77/208 (37.0)	77/198 (38.9)	1.9 (− 6.2, 13.7) 0.460	16/208 (7.7)	21/205 (10.2)	2.5 (− 2.6, 8.6) 0.289	8/208 (3.9)	4/208 (1.9)	− 2.0 (− 5.3, 1.3) 0.206	50/208 (24.0)	60/202 (29.7)	5.7 (− 0.6, 15.3) 0.069	24/208 (11.5)		24/203 (11.8)	0.3 (− 5.5, 6.6) 0.866
Drug use *n*/*n*_disorder_ (%)	24/193 (12.4)	40/140 (28.6)		6/51 (11.8)	12/43 (27.9)		3/17 (17.7)	5/11 (45.5)		13/119 (10.9)	31/98 (31.6)		7/68 (10.3)		16/55 (29.1)	
*n*/*n*_total_ (%)	24/206 (11.7)	40/204 (19.6)	7.9 (3.4, 15.2) 0.002	6/208 (2.9)	12/208 (5.8)	2.9 (− 0.8, 7.0) 0.109	3/208 (1.4)	5/208 (2.4)	1.0 (− 1.8, 4.0) 0.414	13/206 (6.3)	31/205 (15.1)	8.8 (5.6, 15.1) < 0.001	7/208 (3.4)		16/206 (7.8)	4.4 (0.2, 9.0) 0.039

Newcombe confidence intervals and McNemar asymptotic *p* values are based on paired data displayed in Supplementary Material Table S8

Psychiatric disorder includes both primary and additional diagnoses, based on only complete cases (diagnostic assessment at both *T*_1_ and *T*_2_)

^a^RD is risk difference, the difference between the proportions of patients with psychiatric disorder and comorbid substance use at *T*_2_ compared with *T*_1_

^b^Smoking included daily or occasional smokers (*T*_1_ and *T*_2_)

^c^*n*/*n*_disorder_ means that there are (*n*) numbers of patients with comorbid substance use among the patients with the actual psychiatric disorder

^d^*n*/*n*_total_ means that there are (*n*) numbers of patients with comorbid substance use among the total sample of patients

^e^Alcohol use was indicated by answering “yes” to the following questions: *T*_1_: “Do you sometimes drink alcohol presently?”, and *T*_2_: “Have you drunk alcohol during the last four weeks?”

^f^Drug use was indicated by answering “yes” to the question: “Have you ever tried hash, marijuana, or other illicit drugs?” (*T*_1_ and *T*_2_)

### Analysis of associations

Binary linear regression including age or SES as covariate showed no association with persistence of psychiatric disorder, i.e., no confounding effects were found either in the total sample or separately for each gender (Table 5). There was an association between having chronic pain at *T*_1_ and persisting psychiatric disorder for the total sample [RD = 17.2%, CI (7.9, 26.6), *p* < 0.001], and most evident for girls [RD = 25.4%, CI (9.6, 41.2), *p* = 0.002] (Table 4). Associations were also found between smoking and trying illicit drugs and persisting psychiatric disorders among girls [RD = 15.6%, CI (4.1, 27.0), *p* = 0.008, and RD = 18.0%, CI (7.3, 28.6), *p* = 0.001, respectively].

**Table 5:** Binary linear regression with psychiatric disorder at *T*_2_ as dependent variable, and the listed covariates one at a time

Co-variable at *T*_1_	*n*	Any psychiatric disorder *T*_*2*_					
		Co-variable *T*_1_ NO *n* (%)	Co-variable *T*_1_ YES *n* (%)	RD^a^ %	95% CI for RD		*p* value
					Lower	Upper	
Total sample	440						
Chronic pain	438	84/134 (62.7)^b^	243/304 (79.9)	17.2	7.9	26.6	< 0.001
Any somatic disorder	440	269/359 (74.9)	59/81 (72.8)	− 2.1	− 12.8	8.6	0.701
Smoking	304	151/205 (73.7)	84/99 (84.8)	11.2	1.9	20.5	0.018
Alcohol use	440	160/224 (71.4)	168/216 (77.8)	6.3	− 1.8	14.5	0.125
Drug use	437	272/376 (72.3)	54/61 (88.5)	16.2	7.0	25.4	0.001
Age	440			0.105^c^	− 2.39	2.60	0.934
SES	326			0.012^d^	− 2.82	2.80	0.993
Girls	245						
Chronic pain	245	24/43 (55.8)	164/202 (81.2)	25.4	9.6	41.2	0.002
Any somatic disorder	245	149/192 (77.6)	39/53 (73.6)	− 4.0	− 17.3	9.3	0.553
Smoking	178	81/112 (72.3)	58/66 (87.9)	15.6	4.1	27.0	0.008
Alcohol use	245	78/105 (74.3)	110/140 (78.6)	4.3	− 6.5	15.1	0.437
Drug use	244	153/207 (73.9)	34/37 (91.9)	18.0	7.3	28.6	0.001
Age	245			0.200	− 3.21	3.61	0.908
SES	177			0.797	− 4.58	2.98	0.680
Boys	195						
Chronic pain	193	60/91 (65.9)	79/102 (77.5)	11.5	− 1.2	24.2	0.076
Any somatic disorder	195	120/167 (71.9)	20/28 (71.4)	− 0.4	− 18.5	17.7	0.963
Smoking	126	70/93 (75.3)	26/33 (78.8)	3.5	− 13.0	20.1	0.677
Alcohol use	195	82/119 (68.9)	58/76 (76.3)	7.4	− 5.3	20.1	0.253
Drug use	193	119/169 (70.4)	20/24 (83.3)	12.9	− 3.5	29.4	0.124
Age	195			0.881	− 4.78	3.01	0.657
SES	149			0.944	− 3.24	5.13	0.659

^a^RD is risk difference, the difference between the proportions of patients with persistent psychiatric disorder and co-variable present at *T*_1_ compared with patients with persistent psychiatric disorder without present co-variable at *T*_1_

^b^The numbers in this table, for example 84/134 (62.7) and 243/304 (79.9), indicate that among the 134 patients with a psychiatric disorder and no chronic pain at *T*_1_, 84 had a psychiatric disorder at *T*_2_, and among the 304 patients with a psychiatric disorder and chronic pain at *T*_1_, 243 had a psychiatric disorder at *T*_2_

^c^The risk of having a persistent psychiatric disorder increases with 0.105% per one year increase of age

^d^The risk of having a persistent psychiatric disorder increases with 0.012% per one unit change in level of mothers education

## Discussion

This study is one of the few surveys studying the development of psychiatric disorders and comorbidity over time, following a general clinical psychiatric population of adolescents who received standard clinical care. While the general psychiatric morbidity decreased in the course of 3 years, including mood disorders, the rate of anxiety disorders increased, and having more than one psychiatric disorder became more frequent. Altogether, three out of four still had a psychiatric disorder. The most prominent finding was the marked increase of anxiety disorders among girls, accompanied by more psychiatric comorbidity, somatic comorbidity and chronic pain; whereas boys had decreased morbidity overall. Substance use was prevalent among girls with anxiety disorders, while trying illicit drugs clearly involved the most marked increase in both genders. Chronic pain, smoking and trying illicit drugs at the first visit were associated with persisting psychiatric disorders, with highest risk difference for girls.

The reasons for the high rates of persisting disorders may be diverse, both depending on the treatment given and the general vulnerability in the adolescents in this clinical population, who have a high disease burden. In a study of Copeland et al., investigating the cumulative prevalence of psychiatric disorders in young adulthood among 1420 participants assessed between ages 9 and 21, they found that 61.1% met DSM criteria for a well-specified psychiatric disorder by 21 years of age, indicating that many struggle with mental health problems in young adulthood [37]. There is an increase in overall rates of psychiatric disorders in the transition from adolescence to adulthood [7]. Common psychiatric disorders in adolescence are often forerunners and strong predictors of similar disorders in young adulthood, and most young adults with episodes of a psychiatric disorder have had episodes during their teenage years [7, 8, 28, 29], which is in accordance with the findings by Ranøyen et al. in the CAP Survey [38]. Kim-Cohen et al. found that among those who met criteria for a major DSM diagnosis at 26 years, half had a disorder at age 11–15 years, and three out of four before 18 years [39]. The higher frequency among girls overall, and especially in girls with anxiety disorders, is comparable with the earlier research [11, 40]. Results from the Child/Adolescent Anxiety Multimodal Extended Long-Term Study (CAMELS) found that despite receiving high-quality evidence-based treatments for anxiety, only 22% were in stable remission across all 4 years they were assessed, 30% were chronically ill, and 48% experienced relapses [41]. In this study, male gender was associated with increased probability of being in the remission group compared to the relapser group, supporting our finding of higher morbidity among girls. Mood disorders had decreased at follow-up and may have been under-registered in this study. As the course of such disorders is fluctuating, present status may not reflect struggling with periodic disorders.

The female patients seemed to be more prone to develop co-occurring psychiatric disorders. This corresponds well with the previous studies reporting more comorbidity in girls than boys [10, 42]. In our sample, girls had very high rates of psychiatric comorbidity at follow-up, in all diagnostic groups, and highest among those with mood disorders, where more than nine out of ten had an additional psychiatric disorder. All boys with mood disorders also had a comorbid psychiatric disorder, but since there were few boys with mood disorders, the change in frequencies were small. Also, somatic comorbid diagnoses became more frequent for female patients with anxiety disorders. An increased risk of somatic disorders is reported in patients with anxiety disorders [43], independent of gender. Furthermore, adolescents who experience chronic somatic health conditions, are found to be at risk of elevated physiological anxiety symptoms in mid-adolescence [44]. The higher frequency of girls than boys with anxiety disorders in our sample may have influenced the finding of a significant increase only among female patients. Still, the results show consistently that the burden of disease was most prominent among girls.

Overall, there was a decrease in chronic pain for the total sample after 3 years, but an increase among those with anxiety disorders, not surprisingly since chronic pain may be regarded as part of the anxiety disorder. We found large differences in frequency of pain between the genders at *T*_2_. Nearly, nine out of ten girls and five out of ten boys with anxiety disorders had chronic pain, and the frequency of multisite pain was more than three times as high in girls with anxiety disorders compared to boys. A systematic review investigating the epidemiology of chronic pain in children and adolescents from the general population found that pain prevalence was generally higher in girls and increased with age for most pain types [45]. Using data from a large Norwegian population study, Skrove et al. demonstrated higher prevalence of chronic multisite pain among adolescent girls and boys with increasing number of psychiatric symptoms, but with highest rates among girls [46]. In the St. Olav CAP Survey at *T*_1_, 70% of the patients reported chronic pain in addition to a psychiatric disorder [21]. This was a higher frequency than the 44% reported in the general adolescent population in many countries [47] and in our region [22], and underlines the importance of assessing chronic pain among adolescents with psychiatric symptoms and disorders.

Norway appears to be a low-prevalence country when it comes to substance use in the general adolescent population in comparison with other European countries [48]. At *T*_1_, the adolescents reported a higher intake of alcohol, a higher prevalence of smoking, and a four times higher ratio of having tried illicit drugs compared to the general population [24]. In our sample, smoking tended to decrease during follow-up, with no gender differences; whereas, alcohol use did not change substantially overall, but increased for anxiety disorders and ADHD in the total sample and for girls. Some increase was expected, since the age of participants increased from 13–18 years to 16–21 years, and drinking alcohol is more common at these ages. Also, young adults are allowed to buy alcohol in Norway from 18 years. Finding the highest increase among girls with anxiety disorders corresponds well with earlier Norwegian studies [26, 49].

The more surprising result was the significantly increased level of having tried illicit drugs in all diagnostic categories, and especially in the female sample. Getting correct information using self-report on behavior that may be shameful or illegal, may be a challenge. Therefore, the reports on drug use must be interpreted with caution. During adolescence, there is a general increased use of illicit drugs and a possible increased tolerance, which can contribute to an increased incidence 3 years later [50]. Still, associations with the specific psychiatric disorders are relevant. We found highest rates among girls with anxiety disorders; in this group, one out of five had tried illicit drugs. There are inconsistent findings on the association between anxiety disorders and alcohol/drug use in previous studies, some indicating a positive association [51], and other demonstrating negative associations [52]. Opposite to our finding, Turner et al. demonstrated in a review that self-medication with alcohol or drugs for mood and anxiety disorders was associated with male gender [53]. A recent population-based study showed gender-specific substance use patterns among Portuguese adolescents [54]. We found increasing rates of illicit drug use among patients with ADHD for both genders, which corresponds with recent findings from the MTA longitudinal study [55]. Our results and the inconsistent findings between studies indicate the need for further research on gender and disorder-specific substance use in a clinical population.

We examined the possible risk factors associated with the persistence of psychiatric disorders. There was a significant association between having chronic pain at *T*_1_ and persisting psychiatric disorder over 3 years for the total sample, and strongest in the female group. Earlier studies have demonstrated associations between pain in adolescence and mental health problems in young adulthood [18, 19]. Presence of chronic pain in adolescents with psychiatric disorders, especially among girls, is, therefore, important to assess, since these patients seem to be vulnerable for persistent and even increasing psychiatric morbidity. There was also an association between smoking or having tried illicit drugs and persistence of psychiatric morbidity in girls. Socioeconomic status as measured by maternal level of education could not explain the persistence of disorders or the effect of these risk factors, nor could participants’ age.

The strength of the present study is the inclusion of a large clinical sample, providing a high degree of precision in the estimates, and the response rate from *T*_1_ to *T*_2_ was high. Although the attrition rate was high in the initial recruitment, the *T*_1_ sample did not differ in age, gender or reason for referral compared to non-participants. This high attrition rate may still have affected the results, and both severity of symptoms and types of treatment may have played a role in in the continuity of a disorder and for participation at follow-up. The number of participants was low for some diagnostic groups which probably limits the generalizability of the results. Furthermore, due to few participants in some diagnostic groups, especially when examining comorbid chronic pain, somatic disorders and substance use, we chose to merge children with these diagnoses into one larger group.

The psychiatric diagnoses were classified by clinicians, according to the current diagnostic classification systems, and not based on the self-report measures which involves the limitations of less accuracy in establishing psychopathology. However, two different classification systems were used at the two time points, which may have affected prevalence rates and is a possible limitation. Research has shown that the concordance between the two systems can differ across the range of disorders, and with varying concordance within the anxiety disorders [56], and also the diagnostic criteria for hyperkinetic disorder in ICD-10 are more strict than the criteria for ADHD in DSM-IV. In particular, this may have contributed to a higher increase in ADHD diagnosis at follow-up. Beyond that, the ICD-10 and DSM-IV are widely harmonized. The classification process differed between the two time points; At *T*_1_, diagnoses were based on all available clinical information collected by the multi-disciplinary team, and at *T*_2_, the acknowledged K-SADS semi-structured interview was performed as telephone interview. This may also have affected the diagnostic accuracy. K-SADS assesses all psychiatric disorders more systematically, which could have led to reporting more comorbid disorders at *T*_2_, even though secondary disorders were stated on the basis of thorough assessment also at *T*_1_. It is also reasonable to assume that some differences in diagnoses over time might be explained by the different methods and diagnostic procedures between the two time points. There is no well-established definition of chronic pain in children, but the definitions of chronic pain and multisite pain used in this study are widely used in other epidemiologic pain studies, and have been used in the general population in studies from the same area [20, 22]. Still, some information bias cannot be excluded when using self-reports, which could lead to an under- or overestimation of chronic pain and substance use. There was different wording in the question about alcohol use at the two time points. Trying illicit drugs was reported only by one question, and although the same question was used at both time points, this topic may be especially prone to information bias. Using level of maternal education to indicate socioeconomic status may not encircle the entire concept of SES, and furthermore, the SES information was available in a reduced sample, which may not reflect the total study population. Treatment plausibly impact the course of morbidity, and the lack of treatment assessment in this study is a limitation. Since there were more girls than boys among participants compared to non-participants in this study, we may have lost some of the boys with psychiatric disorders.

### Clinical implications

The results of this study bring an important message to clinical practice. Even though clinicians know about mental health challenges in adolescence, the persistence of psychiatric disorders over 3 years from early to late adolescence should be an extra eye-opener, and especially the increased rate of anxiety disorders and comorbidities among girls. The burden of disease in this age group must be acknowledged. In-depth assessment of mental health problems should of course include important risk factors, and asking adolescent patients about pain, uncover smoking habits and illicit drug use seems to be essential, especially for female patients. Providing standard clinical care may not be enough, as these risk factors point to the need for intensified psychiatric treatment to prevent persistence of the psychiatric disorders. Furthermore, long-term clinical follow-up should be considered for adolescents with risk factors.

## Conclusions

Psychiatric morbidity decreased over 3 years in this adolescent clinical sample, including mood disorders, but nevertheless, almost three out of four still had a psychiatric disorder. The high frequency of psychiatric and somatic comorbidity, and chronic pain, indicates generally a high burden of disease, and chronic pain may be seen as part of the complexity of psychiatric disorders, especially anxiety disorders. Female adolescents seemed to have a higher morbidity than male adolescents, with an increased frequency of anxiety disorders after 3 years, and a five–ten times higher prevalence of chronic pain than boys. Chronic pain, smoking and having tried illicit drugs at baseline were factors strongly associated with persistent psychiatric morbidity. Although some differences in diagnoses over time might be explained by the different methods and diagnostic procedures between the two time points, the results indicate the need for addressing the associated factors and include them in a comprehensive follow-up of psychiatric disorders in this age group.

## Electronic supplementary material

Below is the link to the electronic supplementary material.

Supplementary file1 (DOCX 34 kb)
