# Supplementary material for: Psychiatric morbidity, somatic comorbidity and substance use in an adolescent psychiatric population at 3-year follow-up
Source: Eur Child Adolesc Psychiatry. 2020 Jul 15;30(7):1095–112. doi: 10.1007/s00787-020-01602-8 (PMC8295167; doi:10.1007/s00787-020-01602-8)
Supplement: Supplementary file 1 — Supplementary file1 (DOCX 34 kb) [file 787_2020_1602_MOESM1_ESM.docx]

**Supplementary Material**

**Table S1. Age, gender and SES of participants at T_2_ and non-participants at T_2_**

|  | | **Participants T_1_** | | **Participants T_2_** | | **Non-participants T_2_** | |
| --- | --- | --- | --- | --- | --- | --- | --- |
|  | | **(n=717)** | | **(n=570)** | | **(n=147)** | |
| **Age (years) Mean (SD)** | | 15.7 | (1.7) | 15.7 | (1.7) | 15.5 | (1.6) |
| **Gender**  **SES** | **Girls n (%)** | 393 | (54.8) | 324 | (56.8) | 69 | (46.9) |
|  | **Boys n (%)**  **Mean (SD)** | 324  4.7 | (45.2)  (1.7) | 246  4.8 | (43.2)  (1.7) | 78  4.3 | (53.1)  (1.7) |
|  |  |  |  |  |  |  |  |

**Table S2. Age, gender and SES of participants and non-participants in present sample**

|  | | **Diagnostic assessment at T_1_ (n=597)** | | **Participants**  **(n=464)** | | **Non-participants (n=133)** | |
| --- | --- | --- | --- | --- | --- | --- | --- |
| **Age (years) Mean (SD)** | | 15.7 | (1.7) | 15.7 | (1.7) | 15.7 | (1.7) |
| **Gender** | **Girls n (%)** | 321 | (53.8) | 256 | (55.2) | 65 | (48.9) |
|  | **Boys n (%)** | 276 | (46.2) | 208 | (44.8) | 68 | (51.1) |
| **SES** | **Mean (SD)** | 4.7 | (1.7) | 4.8 | (1.7) | 4.4 | (1.6) |

**Table S3. Diagnoses T_1_ of participants and non-participants in present sample**

|  | **Participants (n=464)** | | | | | |  | **Non-participants (n=133)** | | | | | | | | |  |
| --- | --- | --- | --- | --- | --- | --- | --- | --- | --- | --- | --- | --- | --- | --- | --- | --- | --- |
| **Diagnoses at T1** | **Girls (n=256)**  **n (%)** | | **Boys (n=208)**  **n (%)** | | **Total (n=464)**  **n (%)** | |  | | **Girls (n=65)**  **n (%)** | | | **Boys (n=68)**  **n (%)** | | | **Total (n=133)**  **n (%)** | | |
| **Any psychiatric disorder** | 245 | (95.7) | 195 | (93.8) | 440 | (94.8) |  | 62 | | (95.4) | 65 | | (95.6) | 127 | | (95.5) |  |
| **Anxiety disorder** | 96 | (37.5) | 51 | (24.5) | 147 | (31.7) |  | 23 | | (35.4) | 18 | | (26.5) | 41 | | (30.8) |  |
| **Mood disorder** | 82 | (32.0) | 17 | (8.2) | 99 | (21.3) |  | 22 | | (33.8) | 10 | | (14.7) | 32 | | (24.1) |  |
| **ADHD** | 83 | (32.6) | 121 | (58.2) | 204 | (44.0) |  | 23 | | (35.4) | 35 | | (51.5) | 58 | | (43.6) |  |
| **Other psychiatric disorder** | 52 | (20.3) | 68 | (32.7) | 120 | (25.9) |  | 13 | | (20.0) | 30 | | (44.1) | 43 | | (32.3) |  |

**Study of agreement of diagnostic classification:**

The study of agreement between first and second rater was designed as follows: Seven of the interviewers were used as second opinion raters for taped telephone interviews. Each of these seven re-scored four interviews performed by four of the other six interviewers. Hence, the number of re-scored patients were 7x4=28. The design was constructed as shown in Table S4, to be as balanced as possible.

**Table S4. Design of agreement study**

|  |  |  | Second rater | |  |  |  |  |  |  |  |
| --- | --- | --- | --- | --- | --- | --- | --- | --- | --- | --- | --- |
|  |  |  | B | C | E | D | F | G | A |  | Sum |
|  |  |  |  |  |  |  |  |  |  |  |  |
| First rater | B |  |  | 1 | 1 | 1 | 1 | 0 | 0 |  | 4 |
|  | C |  | 1 |  | 1 | 1 | 0 | 1 | 0 |  | 4 |
|  | E |  | 0 | 0 |  | 1 | 1 | 1 | 1 |  | 4 |
|  | D |  | 1 | 1 | 0 |  | 0 | 1 | 1 |  | 4 |
|  | F |  | 1 | 0 | 0 | 1 |  | 1 | 1 |  | 4 |
|  | G |  | 0 | 1 | 1 | 0 | 1 |  | 1 |  | 4 |
|  | A |  | 1 | 1 | 1 | 0 | 1 | 0 |  |  | 4 |
|  |  |  |  |  |  |  |  |  |  |  |  |
|  | Sum |  | 4 | 4 | 4 | 4 | 4 | 4 | 4 |  | 28 |

The diagnostic group variables Depressive, Bipolar, Psychotic, Anxiety, ADHD, and Other were coded as 1(not present), 2 (Partly in remission), and 3 (Present). For the variable Bipolar, the code 2 (partly in remission) was not used. A diagnostic group variable “Any diagnosis” was coded equal to the maximum of the aforementioned codes. A separate variable “suicidal” was coded as “not present” versus “present”.

For each diagnostic group, we quantified the inter-rater reliability between rating 1 and 2 using negative agreement and positive agreement, as recommended by (1). The positive (negative) agreement is the probability that the second rater classifies the patient as with diagnosis (without diagnosis), given that the first rater diagnosed the patient as with diagnosis (without diagnosis). In this setting, we merged “partly” with “no” diagnosis.

Crosstables for diagnostic groups for rating 1 versus rating 2 are shown in Table S5. Negative agreement was generally high, varying from 0.88 to 1.00 for the different disorders. This would be regarded as good to excellent agreement in most settings. The negative agreement was 0 for bipolar and psychotic disorder, and varied from 0.57 to 1.00 for the rest of the disorders.

**Table S5. Interrater reliability for diagnostic groups**

| First rater (rows) and second rater (columns) | | | | |  |  | Specific agreement, Partly merged with No | | |
| --- | --- | --- | --- | --- | --- | --- | --- | --- | --- |
|  |  |  |  |  |  |  |  |  |  |
|  |  |  |  |  |  |  | Positive agreement | | Negative agreement |
|  |  |  |  |  |  |  |  |  |  |
| Depressive disorder presently | | No | Partly | Yes |  |  | 1,000 |  | 1,000 |
|  | No | 23 | 0 | 0 |  |  |  |  |  |
|  | Partly | 3 | 0 | 0 |  |  |  |  |  |
|  | Yes | 0 | 0 | 2 |  |  |  |  |  |
|  |  |  |  |  |  |  |  |  |  |
| Anxiety |  | No | Partly | Yes |  |  | 0,615 |  | 0,884 |
|  | No | 15 | 2 | 1 |  |  |  |  |  |
|  | Partly | 2 | 0 | 1 |  |  |  |  |  |
|  | Yes | 2 | 1 | 4 |  |  |  |  |  |
|  |  |  |  |  |  |  |  |  |  |
| ADHD |  | No | Partly | Yes |  |  | 0,941 |  | 0,974 |
|  | No | 15 | 1 | 0 |  |  |  |  |  |
|  | Partly | 1 | 2 | 0 |  |  |  |  |  |
|  | Yes | 1 | 0 | 8 |  |  |  |  |  |
|  |  |  |  |  |  |  |  |  |  |
| Other |  | No | Partly | Yes |  |  | 0,667 |  | 0,960 |
|  | No | 22 | 1 | 0 |  |  |  |  |  |
|  | Partly | 1 | 0 | 0 |  |  |  |  |  |
|  | Yes | 2 | 0 | 2 |  |  |  |  |  |
|  |  |  |  |  |  |  |  |  |  |
| Any |  | No | Partly | Yes |  |  | 0,929 |  | 0,929 |
|  | No | 7 | 4 | 0 |  |  |  |  |  |
|  | Partly | 1 | 1 | 0 |  |  |  |  |  |
|  | Yes | 0 | 2 | 13 |  |  |  |  |  |

1. de Vet HC, Mokkink LB, Terwee CB et al. Clinicians are right not to like Cohen's kappa. BMJ 2013; 346: f2125.

**Table S6. Paired data of the presence of disorders and psychiatric comorbidity at T_1_ and T_2_**

|  | | | **Disorder/comorbidity T_1_ NO** | | | | | **Disorder/comorbidity T_1_ YES** | | | |
| --- | --- | --- | --- | --- | --- | --- | --- | --- | --- | --- | --- |
| **Total sample (n=464)** | | **Disorder/co-morbidity T_2_ NO** | | | | **Disorder/co-morbidity T_2_ YES** | | **Disorder/co-morbidity T_2_ NO** | | **Disorder/co-morbidity T_2_ YES** | |
| Any psychiatric disorder | | | | 17 | 7 | | 112 | | 328 | |  |
| Anxiety disorder | | | | 207 | 110 | | 71 | | 76 | |  |
| Mood disorder | | | | 324 | 41 | | 63 | | 36 | |  |
| ADHD | | | | 215 | 44 | | 56 | | 148 | |  |
| Other psychiatric disorder | | | | 297 | 47 | | 60 | | 60 | |  |
| Any psychiatric disorder and comorbid psychiatric disorder | | | | 226 | 108 | | 69 | | 61 | |  |
| Anxiety disorder and comorbid psychiatric disorder | | | | 292 | 113 | | 36 | | 23 | |  |
| Mood disorder and comorbid psychiatric disorder | | | | 359 | 60 | | 32 | | 13 | |  |
| ADHD and comorbid psychiatric disorder | | | | 322 | 66 | | 41 | | 34 | |  |
| Other psychiatric disorder and comorbid psychiatric disorder | | | | 339 | 55 | | 39 | | 31 | |  |
| **Girls (n=256)** | | | |  |  | |  | |  | |  |
| Any psychiatric disorder | | | | 8 | 3 | | 57 | | 188 | |  |
| Anxiety disorder | | | | 76 | 84 | | 37 | | 59 | |  |
| Mood disorder | | | | 141 | 33 | | 49 | | 33 | |  |
| ADHD | | | | 145 | 27 | | 19 | | 64 | |  |
| Other psychiatric disorder | | | | 177 | 27 | | 29 | | 23 | |  |
| Any psychiatric disorder and comorbid psychiatric disorder | | | | 104 | 84 | | 36 | | 32 | |  |
| Anxiety disorder and comorbid psychiatric disorder | | | | 125 | 95 | | 22 | | 14 | |  |
| Mood disorder and comorbid psychiatric disorder | | | | 169 | 49 | | 25 | | 13 | |  |
| ADHD and comorbid psychiatric disorder | | | | 181 | 45 | | 15 | | 14 | |  |
| Other psychiatric disorder and comorbid psychiatric disorder | | | | 197 | 36 | | 14 | | 9 | |  |
| **Boys (n=208)** |  | | | |  | |  | |  | |  |
| Any psychiatric disorder | | | | 9 | 4 | | 55 | | 140 | |  |
| Anxiety disorder | | | | 131 | 26 | | 34 | | 17 | |  |
| Mood disorder | | | | 183 | 8 | | 14 | | 3 | |  |
| ADHD | | | | 70 | 17 | | 37 | | 84 | |  |
| Other psychiatric disorder | | | | 120 | 20 | | 31 | | 37 | |  |
| Any psychiatric disorder and comorbid psychiatric disorder | | | | 122 | 24 | | 33 | | 29 | |  |
| Anxiety disorder and comorbid psychiatric disorder | | | | 167 | 18 | | 14 | | 9 | |  |
| Mood disorder and comorbid psychiatric disorder | | | | 190 | 11 | | 7 | | 0 | |  |
| ADHD and comorbid psychiatric disorder | | | | 141 | 21 | | 26 | | 20 | |  |
| Other psychiatric disorder and comorbid psychiatric disorder | | | | 142 | 19 | | 25 | | 22 | |  |

**Table S7. Paired data of the presence of disorders and somatic comorbidity at T_1_ and T_2_**

|  | **Comorbidity T_1_ NO** | | | | | **Comorbidity T_1_ YES** | | | |
| --- | --- | --- | --- | --- | --- | --- | --- | --- | --- |
| **Total sample (n=464)** | **Comorbidity T_2_ NO** | | | **Comorbidity T_2_ YES** | | **Comorbidity T_2_ NO** | | **Comorbidity T_2_ YES** | |
| Any psychiatric disorder and comorbid somatic disorder | | 322 | 61 | | 57 | | 24 | |  |
| Anxiety disorder and comorbid somatic disorder | | 381 | 50 | | 24 | | 9 | |  |
| Mood disorder and comorbid somatic disorder | | 424 | 19 | | 16 | | 5 | |  |
| ADHD and comorbid somatic disorder | | 393 | 39 | | 24 | | 7 | |  |
| Other psychiatric disorder and comorbid somatic disorder | | 420 | 24 | | 17 | | 3 | |  |
| Any psychiatric disorder and chronic pain | | 119 | 37 | | 110 | | 188 | |  |
| Anxiety disorder and chronic pain | | 257 | 98 | | 59 | | 49 | |  |
| Mood disorder and chronic pain | | 343 | 41 | | 49 | | 30 | |  |
| ADHD and chronic pain | | 274 | 51 | | 63 | | 71 | |  |
| Other psychiatric disorder and chronic pain | | 349 | 41 | | 44 | | 29 | |  |
| Any psychiatric disorder and multisite pain | | 241 | 41 | | 69 | | 99 | |  |
| Anxiety disorder and multisite pain | | 320 | 73 | | 38 | | 30 | |  |
| Mood disorder and multisite pain | | 374 | 33 | | 38 | | 18 | |  |
| ADHD and multisite pain | | 352 | 37 | | 40 | | 30 | |  |
| Other psychiatric disorder and multisite pain | | 397 | 27 | | 21 | | 18 | |  |
| **Girls (n=256)** | |  |  | |  | |  | |  |
| Any psychiatric disorder and comorbid somatic disorder | | 164 | 39 | | 35 | | 18 | |  |
| Anxiety disorder and comorbid somatic disorder | | 189 | 43 | | 17 | | 7 | |  |
| Mood disorder and comorbid somatic disorder | | 222 | 17 | | 12 | | 5 | |  |
| ADHD and comorbid somatic disorder | | 219 | 22 | | 11 | | 3 | |  |
| Other psychiatric disorder and comorbid somatic disorder | | 230 | 15 | | 8 | | 3 | |  |
| Any psychiatric disorder and chronic pain | | 119 | 37 | | 110 | | 188 | |  |
| Anxiety disorder and chronic pain | | 94 | 80 | | 35 | | 46 | |  |
| Mood disorder and chronic pain | | 152 | 34 | | 40 | | 29 | |  |
| ADHD and chronic pain | | 160 | 26 | | 21 | | 48 | |  |
| Other psychiatric disorder and chronic pain | | 190 | 24 | | 25 | | 17 | |  |
| Any psychiatric disorder and multisite pain | | 241 | 41 | | 69 | | 99 | |  |
| Anxiety disorder and multisite pain | | 132 | 65 | | 27 | | 29 | |  |
| Mood disorder and multisite pain | | 173 | 29 | | 35 | | 18 | |  |
| ADHD and multisite pain | | 183 | 27 | | 23 | | 22 | |  |
| Other psychiatric disorder and multisite pain | | 211 | 22 | | 11 | | 12 | |  |
| **Boys (n=208)** | |  |  | |  | |  | |  |
| Any psychiatric disorder and comorbid somatic disorder | | 158 | 22 | | 22 | | 6 | |  |
| Anxiety disorder and comorbid somatic disorder | | 192 | 7 | | 7 | | 2 | |  |
| Mood disorder and comorbid somatic disorder | | 202 | 2 | | 4 | | 0 | |  |
| ADHD and comorbid somatic disorder | | 174 | 17 | | 13 | | 4 | |  |
| Other psychiatric disorder and comorbid somatic disorder | | 190 | 9 | | 9 | | 0 | |  |
| Any psychiatric disorder and chronic pain | | 82 | 20 | | 51 | | 47 | |  |
| Anxiety disorder and chronic pain | | 163 | 18 | | 24 | | 3 | |  |
| Mood disorder and chronic pain | | 191 | 7 | | 9 | | 1 | |  |
| ADHD and chronic pain | | 114 | 25 | | 42 | | 23 | |  |
| Other psychiatric disorder and chronic pain | | 159 | 17 | | 19 | | 12 | |  |
| Any psychiatric disorder and multisite pain | | 150 | 9 | | 21 | | 18 | |  |
| Anxiety disorder and multisite pain | | 188 | 8 | | 11 | | 1 | |  |
| Mood disorder and multisite pain | | 201 | 4 | | 3 | | 0 | |  |
| ADHD and multisite pain | | 169 | 10 | | 17 | | 8 | |  |
| Other psychiatric disorder and multisite pain | | 186 | 5 | | 10 | | 6 | |  |

**Table S8. Paired data of the presence of disorders and substance use at T_1_ and T_2_**

|  | **Substance use T_1_ NO** | | **Substance use T_1_ YES** | |
| --- | --- | --- | --- | --- |
| **Total sample (n=464)** | **Substance use T_2_ NO** | **Substance use T_2_ YES** | **Substance use T_2_ NO** | **Substance use T_2_ YES** |
| Any psychiatric disorder and smoking | 193 | 32 | 48 | 50 |
| Anxiety disorder and smoking | 344 | 40 | 17 | 12 |
| Mood disorder and smoking | 387 | 22 | 23 | 7 |
| ADHD and smoking | 333 | 25 | 28 | 17 |
| Other psychiatric disorder and smoking | 368 | 26 | 18 | 7 |
| Any psychiatric disorder and current alcohol use | 161 | 80 | 82 | 127 |
| Anxiety disorder and current alcohol use | 301 | 88 | 39 | 30 |
| Mood disorder and current alcohol use | 367 | 30 | 49 | 16 |
| ADHD and current alcohol use | 302 | 66 | 32 | 55 |
| Other psychiatric disorder and current alcohol use | 356 | 46 | 40 | 17 |
| Any psychiatric disorder and drug use | 328 | 67 | 15 | 45 |
| Anxiety disorder and drug use | 386 | 59 | 8 | 10 |
| Mood disorder and drug use | 414 | 31 | 14 | 4 |
| ADHD and drug use | 380 | 44 | 8 | 24 |
| Other psychiatric disorder and drug use | 408 | 39 | 9 | 6 |
| **Girls (n=256)** |  |  |  |  |
| Any psychiatric disorder and smoking | 102 | 19 | 32 | 34 |
| Anxiety disorder and smoking | 170 | 32 | 11 | 12 |
| Mood disorder and smoking | 194 | 19 | 20 | 5 |
| ADHD and smoking | 198 | 10 | 18 | 8 |
| Other psychiatric disorder and smoking | 215 | 14 | 9 | 5 |
| Any psychiatric disorder and current alcohol use | 70 | 44 | 52 | 86 |
| Anxiety disorder and current alcohol use | 130 | 69 | 26 | 28 |
| Mood disorder and current alcohol use | 170 | 27 | 42 | 15 |
| ADHD and current alcohol use | 179 | 34 | 13 | 27 |
| Other psychiatric disorder and current alcohol use | 194 | 28 | 23 | 11 |
| Any psychiatric disorder and drug use | 173 | 43 | 8 | 29 |
| Anxiety disorder and drug use | 194 | 49 | 4 | 8 |
| Mood disorder and drug use | 213 | 27 | 12 | 3 |
| ADHD and drug use | 209 | 24 | 7 | 13 |
| Other psychiatric disorder and drug use | 223 | 25 | 4 | 4 |
| **Boys (n=208)** |  |  |  |  |
| Any psychiatric disorder and smoking | 91 | 13 | 16 | 16 |
| Anxiety disorder and smoking | 174 | 8 | 6 | 0 |
| Mood disorder and smoking | 193 | 3 | 3 | 2 |
| ADHD and smoking | 135 | 15 | 10 | 9 |
| Other psychiatric disorder and smoking | 153 | 12 | 9 | 2 |
| Any psychiatric disorder and current alcohol use | 91 | 36 | 30 | 41 |
| Anxiety disorder and current alcohol use | 171 | 19 | 13 | 2 |
| Mood disorder and current alcohol use | 197 | 3 | 7 | 1 |
| ADHD and current alcohol use | 123 | 32 | 19 | 28 |
| Other psychiatric disorder and current alcohol use | 162 | 18 | 17 | 6 |
| Any psychiatric disorder and drug use | 155 | 24 | 7 | 16 |
| Anxiety disorder and drug use | 192 | 10 | 4 | 2 |
| Mood disorder and drug use | 201 | 4 | 2 | 1 |
| ADHD and drug use | 171 | 20 | 1 | 11 |
| Other psychiatric disorder and drug use | 185 | 14 | 5 | 2 |
